# Rethinking malaria elimination: a perspective on challenges and solutions in Angola

**DOI:** 10.1186/s12936-025-05398-3

**Published:** 2025-07-02

**Authors:** Edmilson Serra Domingos

**Affiliations:** Sagrada Esperança Clinic, Av. Murtala Mohammed, 298 Luanda, Angola

**Keywords:** Malaria elimination, Public health, Vector control, Child mortality, Preventive treatment, Healthcare system challenges, Angola, Antimalarial resistance

## Abstract

**Background:**

Multiple challenges hinder malaria control in Angola, including climatic variability, ineffective vector control, population displacement, socioeconomic inequalities, and increasing resistance to anti-malarial drugs and insecticides. These barriers have been further exacerbated by the COVID-19 pandemic, disrupting healthcare services and reversing prior gains. Despite a 36% reduction in malaria mortality since 2000, Angola remains off track to meet the Global Technical Strategy (GTS) targets for 2025, with no significant progress recorded in reducing malaria mortality between 2015 and 2023.

**Perspectives:**

This paper analyses Angola’s malaria landscape, emphasizing that persistent healthcare system weaknesses such as financial instability, workforce shortages, poor disease surveillance, and regional disparities in intervention coverage, necessitate urgent, tailored responses. Drawing from lessons learned in successful malaria elimination programmes in Cabo Verde, Algeria, China, and the recent WHO recommendations, the study recommends the implementation of three integrated strategies: (i) mass drug administration, to rapidly reduce transmission and help consolidate malaria control; (ii) intermittent preventive treatment for school-age children, to protect a high-risk yet often overlooked population; and (iii) post-hospitalization malaria prevention to decrease readmissions and mortality linked to severe malaria episodes.

**Conclusion:**

Achieving malaria elimination or a substantial reduction in Angola’s disease burden demands increased political commitment, sustainable financing, professional capacity building, and rigorous monitoring. A coordinated, evidence-based approach, aligned with WHO recommendations and tailored to Angola’s epidemiological context, is essential to overcoming barriers and accelerating progress toward the 2030 malaria elimination goal.

## Background

Malaria remains the leading cause of mortality in Angola, representing one of the country’s most pressing public health challenges, particularly affecting children under five and pregnant women [1]. *Plasmodium falciparum*, the most lethal malaria-causing protozoan, predominates and sustains high transmission rates across the country. Between 2010 and 2021, Angola reported approximately 127,507 malaria-related deaths [2, 3], and in 2023, the country ranked second in the Central African region, accounting for 13% of malaria-related mortality and 14% of new cases [4].

Although significant progress has been made, including a 36% reduction in malaria mortality since 2000 and a decrease in morbidity from 26.6 to 15% between 2000 and 2018 [3, 5], several challenges persist. The COVID-19 pandemic severely disrupted malaria services, and Angola was among the five countries with the highest increase in malaria cases between 2019 and 2021, registering an additional 1.4 million cases [2]. Interestingly, regions with higher malaria prevalence reported lower COVID-19 incidence, suggesting a potential protective effect, although this association remains speculative [6].

Multiple challenges hinder malaria control in Angola, including climatic variability, seasonal transmission shifts, ineffective vector control, internal population displacement, growing resistance to anti-malarial drugs and insecticides, and persistent socioeconomic inequalities [2, 5]. These challenges are especially pronounced in rural areas, where access to healthcare and preventive measures remains limited.

Angola, alongside other member states of the Southern African Development Community (SADC)—including Botswana, Eswatini, Mozambique, Namibia, South Africa, Zambia, and Zimbabwe—is implementing coordinated malaria control strategies with the goal of achieving elimination by 2030. Despite coordinated efforts under regional initiatives such as the Elimination 8 (E8) and Angola’s ambition to transition toward pre-elimination [1, 7], the country remains off track to meet the Global Technical Strategy (GTS) for Malaria targets, which call for a 75% reduction in incidence and mortality by 2025.

Given this scenario, this paper analyses the persistent challenges hampering malaria elimination in Angola, critically evaluate existing interventions, and propose targeted strategies to accelerate progress. By aligning national actions with Sustainable Development Goal (SDG) 3—Good Health and Well-being, particularly target 3.3 to end malaria epidemics by 2030, this perspective highlights the urgent need for innovative, coordinated, and sustainable interventions adapted to Angola’s specific epidemiological context.

### Key challenges in an endemic context

Malaria transmission in Angola is heavily influenced by climatic factors, being unviable at temperatures below 16 °C or above 40 °C. The country’s equatorial, tropical, and subtropical zones create highly favourable conditions for year-round transmission [8]. However, disease burden varies across regions, with higher intensity observed in areas with elevated humidity and precipitation [9, 10].

Beyond environmental conditions, multiple socioeconomic and healthcare system weaknesses exacerbate the malaria crisis. Malaria remains a major contributor to child mortality, particularly in impoverished communities where limited education, poor sanitation, lack of access to clean water and electricity, and improper use of mosquito nets sustain high transmission rates. Pregnant women, especially in rural areas, are particularly vulnerable, often resulting in low birth weight and elevated infant mortality [3, 5, 11].

Angola’s population of approximately 36 million faces serious structural challenges that constrain effective malaria response efforts. These challenges include [5, 12–15]:

- Inconsistent and inadequate financial investments, limiting the sustainability of malaria control programmes.
- Restricted access to healthcare, particularly in rural and remote areas.
- Growing mosquito resistance to insecticides and misuse of insecticide-treated nets.
- Parasite resistance to anti-malarial drugs, threatening treatment effectiveness.
- Insufficient indoor residual spraying, leaving vulnerable communities unprotected.
- Incomplete implementation of the malaria vaccine, restricting its potential impact.
- Coexistence with neglected tropical diseases and the lingering effects of COVID-19 on health services.
- Internal migration, complicating surveillance and intervention efforts.
- Low health literacy and delays in seeking treatment, often driven by cultural and economic barriers.

The persistent burden of neglected tropical diseases (NTDs) in Angola presents a critical barrier to effective malaria control, particularly in impoverished and underserved regions. Limited financial resources, competing health priorities, and inadequate infrastructure such as poor sanitation and restricted access to safe water undermine the reach and sustainability of malaria interventions. Furthermore, co-infections with NTDs may compromise immune responses, increasing vulnerability to severe malaria, especially among children under five, thereby exacerbating both incidence and mortality [16].

Additionally, the COVID-19 pandemic significantly disrupted malaria control efforts in low- to middle-income countries like Angola. Lockdown measures led to health service interruptions, restricted geographic accessibility, limited transportation, and compromised malaria control supply chains [17]. Recent evidence from a low-endemic area in Angola indicates a 10.2% increase in malaria-related mortality in 2021, primarily due to reduced hospital access, delayed treatment-seeking, and fear of COVID-19 infection [18]. Moreover, a study conducted in Luanda, although based on a limited sample, highlighted that co-infection between vector-borne diseases and COVID-19 further strained health systems, emphasizing the need for improved screening and patient management [19]. As healthcare services normalized post-pandemic, malaria mortality rates declined, highlighting systemic vulnerabilities and reinforcing the urgent need for resilient strategies in malaria control during public health emergencies.

These complex and intersecting challenges reinforce that malaria control is more effective when multiple preventive methods are combined rather than relying on single interventions [20]. In Angola, addressing these vulnerabilities requires an integrated, well-funded, and evidence-based strategy, focused on expanding healthcare access, improving vector control, and strengthening health systems to sustain progress toward malaria elimination.

### Critical analysis of malaria elimination in Angola

Angola’s malaria control strategy has incorporated key interventions over the years, including intermittent preventive treatment in pregnancy (IPTp), introduced in 2006, and distribution of insecticide-treated nets (ITNs), including long-lasting insecticidal nets (LLINs), currently reaching about 30% of the population [1].

Between 2017 and 2019, a community-based initiative, known as ADECOS (Community and Health Development Agents), demonstrated promising results by conducting over 100,000 rapid diagnostic tests, treating approximately 40,000 malaria cases, and promoting health education campaigns [1]. Policy shifts addressing chloroquine resistance led Angola to adopt artemisinin-based combination therapy (ACT) as the first-line treatment for uncomplicated malaria by 2007/08 [1]. More recently, in 2023, the country distributed over 1.3 million ACT, 1.4 million rapid diagnostic tests, and carried out large-scale mosquito net distributions [4].

Despite these efforts, national progress has remained limited. While the southern provinces have witnessed a decline in malaria incidence, Angola, overall, continues to face persistent challenges that slow its path toward elimination. A central obstacle is the lack of financial autonomy and effective resource management, a recurring challenge across Southern African Development Community (SADC) countries, particularly as external funding diminishes [7].

Malaria control efforts in these countries, including Angola, rest on six fundamental pillars: epidemiological surveillance, vector control, case diagnosis and treatment, behavioral change, and operational research [7]. Given Angola’s growing population and persistent transmission rates, expanded and sustainable funding is critical to scale up effective interventions.

Notably, Angola has achieved significant progress in malaria control, particularly in reducing child mortality among children under five years of age [5]. Nonetheless, persistent shortages in the healthcare workforce, especially in rural areas, continue to undermine intervention efforts. These challenges are compounded by logistical constraints, including hospital-level infrastructural barriers, inconsistent access to malaria diagnostics and treatments, and frequent disruptions in supply chains, all of which contribute to inefficiencies in service delivery, particularly in underserved regions [4, 21]. Furthermore, weak health information systems, characterized by gaps in data collection, processing, and real-time reporting, hinder strategic decision-making and the implementation of tailored interventions [21].

Moreover, vector control strategies remain insufficiently implemented, with low coverage and inconsistent application across provinces, limiting their overall impact. The absence of a standardized, evidence-based approach, considering climatic variations and vector resistance patterns, further weakens malaria control efforts [22].

Lessons from successful malaria elimination programmes in Cabo Verde and Algeria emphasize the importance of integrating sustainable strategies, such as residual spraying and larval control, which were key to achieving elimination [2, 23–25]. Similarly, China successfully eliminated malaria through an integrated approach, establishing disease control stations in multiple regions to enable continuous epidemiological surveillance and rapid response. Additionally, the country implemented free treatment distribution, encouraged local governments to set annual case reduction targets, and mobilized communities to actively participate in prevention efforts. Preventive treatments were strategically administered to targeted populations during key transmission periods. These measures, combined with rigorous vector control and extensive health education campaigns, were essential to the country’s malaria eradication success [2, 26, 27].

Nevertheless, rising insecticide resistance and documented therapeutic failures of Artemether-Lumefantrine in 2015, 2019, and 2021 highlight the fragility of current gains and the urgent need for continuous monitoring of drug efficacy [4].

In 2023, no significant progress was observed in reducing malaria mortality compared to 2015 [4], reaffirming that Angola is currently off-track to meet the Global Technical Strategy (GTS) goals of a 75% reduction in malaria incidence and mortality by 2025. Without major reinforcements to control strategies, sustainable progress toward elimination remains out of reach.

Addressing these realities requires scaling-up proven interventions, strengthening programmatic and operational capacities, and adopting innovative solutions adapted to Angola’s complex context.

### Proposed interventions for malaria control in Angola

Angola stands at a critical crossroads in the fight against malaria. This paper urgently calls for the adoption of mass drug administration, intermittent preventive treatment for school-age children, and post-hospitalization malaria prevention to close existing intervention gaps and accelerate the elimination efforts.

Firstly, mass drug administration (MDA) involves administering a full course of antimalarial treatment to all individuals within a target population, regardless of infection status, aiming to eliminate existing infections and prevent new ones. This intervention has demonstrated short-term efficacy, although evidence on its long-term effects remains limited [29, 30]. Moreover, concerns persist regarding the sustainability of the intervention and the potential emergence of drug resistance [30, 31]. Despite these limitations, MDA can offer strategic benefits in low- and middle-income settings such as Angola, particularly in contexts of overburdened health systems, geographic areas with limited access to care, complex emergencies, or recurring malaria outbreaks, where rapid reductions in incidence and mortality are critical [2, 32].

Beyond its use in epidemic control, MDA may also serve as a complementary tool in pre-elimination settings, especially in isolated low-transmission areas, when combined with strategies, such as indoor residual spraying (IRS) or seasonal malaria chemoprevention integrated with seasonal malaria vaccination (30). China’s experience exemplifies MDA’s potential: between 1949 and 1999, presumptive treatment was administered to both symptomatic and asymptomatic individuals, and radical MDA campaigns were conducted, later being targeted to specific regions during the elimination phase [27].

In Angola, MDA could be considered to consolidate control in less endemic southern provinces [1] or be targeted toward previously identified high-transmission areas [33], particularly where hospital care is limited. However, MDA should not be viewed as a substitute for vector control measures. Its impact on transmission is generally temporary, requiring repeated rounds for sustained effect [34], and careful consideration must be given to the logistical and sustainability challenges involved.

In parallel, malaria is a major contributor to anaemia among school-age children in sub-Saharan Africa, including Angola [35]. Intermittent Preventive Treatment for School-Age Children (IPTsc) involves a periodic administration of anti-malarial drugs to children aged 5–15 years. This intervention can reduce the malaria burden, which is closely associated with severe anaemia not only in children under five but also in those aged 5–15, particularly in hyperendemic areas, such as northern Angola, where heavy rainfall intensifies transmission [1, 35]. Given the high transmission intensity and the limited inclusion of school-age children in current malaria strategies, Angola presents a strong case for the adoption of IPTsc. This intervention is supported by recent evidence from Africa [36] and could be integrated into national and provincial malaria control programmes, while also being aligned with nutritional initiatives to strengthen efforts to reduce anaemia among school-age children.

Notably, lessons from Tanzania’s IPTsc programme which demonstrated significant coverage benefits and a reduction in malaria-related school absenteeism can inform implementation in Angola and similar settings. Key components included cluster-randomized drug administration and strong community engagement [37].

Furthermore, Post-Hospitalization Malaria Prevention (PHMP) may represent a valuable strategy in the Angolan context, given the high rate of malaria-related readmissions and the frequent occurrence of complications, such as severe anaemia among vulnerable groups [38]. This intervention has the potential to improve quality of life in affected children, reduce malaria-related mortality, and mitigate the economic burden on both families and the national health system. The World Health Organization (WHO) recommends a repeated course of anti-malarial therapy following discharge, and several randomized controlled trials conducted in African settings have demonstrated its efficacy in reducing mortality and hospital readmissions over a six-month period [39]. Although the protective effect was restricted to the intervention period [40], this does not undermine the value of the protection provided during that timeframe. Significant progress has been reported in Kenya, Malawi, Uganda, and Benin [39], offering valuable insights for Angola and countries facing similar epidemiological and health system challenges. In Malawi, for instance, reminder systems were explored to ensure adherence to the monthly post-discharge anti-malarial regimen [40]. Implementation of this strategy can follow a facility-based model, where caregivers return to health facilities at scheduled intervals to receive anti-malarial medication. However, in Angola, this model may face adherence barriers due to transportation constraints. Alternatively, a community-based approach in which community health workers deliver medication directly to households [33] would require a robust and well-supported community health infrastructure. Therefore, in Angola, the success of PHMP will depend on the development of sustainable policies and financing mechanisms, effective implementation and monitoring systems, and further research into its cost-effectiveness and integration with existing malaria control strategies.

### Recommendations

It is recommended that the Angolan Ministry of Health adopt the WHO guidelines for malaria control [2, 4], with a particular focus on the measures outlined in Table 1. To maximize the effectiveness of these strategies, it is essential to:

- Increase funding for malaria programmes, ensuring long-term financial sustainability;
- Enhance training for healthcare professionals, strengthening diagnostic and treatment capacity;
- Strengthen vector control efforts, including improved insecticide resistance management and targeted spraying campaigns;
- Promote community engagement, encouraging participation in malaria prevention and early treatment-seeking behavior.

**Table 1:** Proposed interventions for malaria control in Angola (adapted from WHO guidelines). Source: Author own elaboration

Recommendation	Description
Mass Drug Administration (MDA)	This strategy involves administering a full course of antimalarial treatment to all individuals within a target population, regardless of infection status, aiming to eliminate existing infections and prevent new ones. Similar programmes in China reduced malaria burden by 99.31% (27), making this a promising approach for Angola
Intermittent Preventive Treatment for School-Age Children	In areas with moderate to high transmission, periodic administration of antimalarial drugs to children aged 5–15 years can help reduce disease burden without compromising protection for children under five, who are at higher risk of severe complications (2). Additionally, seasonal preventive therapy is a cost-effective intervention for children, particularly in regions where malaria cases spike during rainy seasons (4)
Post-Hospitalization Malaria Prevention	Children with severe anaemia discharged from hospitals in moderate and high-transmission areas should continue preventive antimalarial treatment to avoid reinfection, significantly reducing childhood morbidity and mortality (24)

While Angola is advancing in vaccine implementation, eliminating mosquito breeding grounds and improving basic sanitation remain critical to controlling malaria transmission.

The larval source management is not a panacea but rather an intervention that can be efficient when complemented and integrated with broader malaria control strategy [28]. Evidence from Cabo Verde's elimination programme demonstrates that systematic mapping, monitoring, and treatment of anthropogenic breeding sites particularly when seasonally intensified can contribute meaningfully to reducing transmission. A coordinated, multi-sectoral approach is needed, ensuring integrated and sustained interventions across ministries. Poor sanitation continues to be a major barrier in the fight against malaria and other neglected tropical diseases, reinforcing the urgent need for stronger political commitment to implement effective control measures.

Finally, to support Angola’s elimination efforts, this paper proposes three high-impact strategies: Mass Drug Administration (MDA) to rapidly reduce transmission; Intermittent Preventive Treatment for School-Age Children (IPTsc) to address an often-overlooked risk group; and Post-Hospitalization Malaria Prevention (PHMP) to mitigate post-discharge morbidity and mortality associated with severe malaria.

## Conclusion

The strategies adopted in Angola for malaria control present opportunities for improvement but are constrained by financial, structural, and operational challenges. To achieve sustained progress, malaria programmes must be comprehensive, continuous, integrated, robust, well-funded, rigorous, efficient, and sustainable, with a strong focus on measurable outcomes and meticulous monitoring. Addressing healthcare workforce shortages, resource mismanagement, and intervention gaps is essential to strengthening malaria response efforts. Professional training, improved resource allocation, and adoption of innovative strategies, including enhanced vector control and targeted research on intervention effectiveness, are key priorities. By implementing these measures, Angola can accelerate its path toward malaria elimination, aligning with the Global Technical Strategy (GTS) for Malaria and Sustainable Development Goal 3 (Good Health and Well-being). However, achieving this goal will require increased financial investment, evidence-based policymaking, and stronger multi-sectoral collaboration to overcome persistent barriers and drive sustainable change.

## Data Availability

No datasets were generated or analysed during the current study.

## Abbreviations

SADC: Southern African Development Community
E8: Elimination 8
SDG: Sustainable Development Goal
MDA: Mass Drug Administration
IPTp: Intermittent Preventive Treatment in pregnancy
ITNs: Insecticide-treated nets
LLINs: Long-lasting insecticidal nets
ACT: Artemisinin-based combination therapy
ADECOS: Community and Health Development Agents
GTS: Global Technical Strategy
WHO: World Health Organization
IRS: Indoor residual spraying
IPTsc: Intermittent Preventive Treatment for school-age children
PHMP: Post-Hospitalization Malaria Prevention
